# Method for the Determination of Ammonia in Mainstream Cigarette Smoke Using Ion Chromatography

**DOI:** 10.1371/journal.pone.0159126

**Published:** 2016-07-14

**Authors:** Christina Vaughan Watson, June Feng, Liza Valentin-Blasini, Rayman Stanelle, Clifford H. Watson

**Affiliations:** Centers for Disease Control and Prevention, National Center for Environmental Health, Division of Laborarory Sciences, Tobacco and Volatiles Branch, Atlanta, Georgia, United States of America; Waseda University, JAPAN

## Abstract

Ammonia in mainstream smoke is present in both the particulate and vapor phases. The presence of ammonia in the cigarette filler material and smoke is of significance because of the potential role ammonia could have in raising the “smoke pH.” An increased smoke pH could shift a fraction of total nicotine to free-base nicotine, which is reportedly more rapidly absorbed by the smoker. Methods measuring ammonia in smoke typically employ acid filled impingers to trap the smoke. We developed a fast, reliable method to measure ammonia in mainstream smoke without the use of costly and time consuming impingers to examine differences in ammonia delivery. The method uses both a Cambridge filter pad and a Tedlar bag to capture particulate and vapor phases of the smoke. We quantified ammonia levels in the mainstream smoke of 50 cigarette brands from 5 manufacturers. Ammonia levels ranged from approximately 1μg to 23μg per cigarette for ISO smoking conditions and 38μg to 67μg per cigarette for Canadian intense smoking conditions and statistically significance differences were observed between brands and manufacturers. Our findings suggest that ammonia levels vary by brand and are higher under Canadian intense smoking conditions.

## Introduction

The mainstream cigarette smoke aerosol is a complex mixture of chemicals in a dynamic state, with some analytes of interest existing in both the particulate and vapor phases. Ammonia in mainstream smoke is such an analyte, being both volatile and water soluble, it is present in both phases. Ammonia is a common cigarette additive [1–2], though the reasons for ammonia addition to the tobacco blend are often debated [3–4]. Ammonia is reportedly used as a flavorant and in the manufacture of the reconstituted tobacco sheet [5–6]. According to internal industry documents, “ammonia technology” was a result of attempts by Philip Morris to engineer a sturdier reconstituted sheet. The introduction of ammonia technology was noted to coincide with a sudden increase in market share of the Marlboro brand in the 1960’s [3,7]. Competitors extensively researched the Marlboro product and concluded that an increase in “smoke pH” resulted from the introduction of “ammonia technology” [8]. Most nicotine in mainstream smoke is in the non-volatile, protonated form. However, slight increases in pH can cause more nicotine to be available in the deprotonated, free base form [9–10]. Free-base nicotine is thought to be more rapidly available to the smoker through two mechanisms: 1) being more lipophilic, it can cross cell membranes more efficiently and 2) being more volatile, the free-base form should be in the vapor phase, eliminating the need to diffuse from smoke particles [9,11]. Additionally, ammonia can also react acids and aldehydes to smooth the smoke [12]. Ammonia causes eye and respiratory tract irritation [13] and is included as a respiratory toxicant on the U.S. Food and Drug Administration’s list of harmful and potentially harmful constituents of tobacco smoke [14].

Existing data for ammonia in mainstream smoke indicates there is a large difference between amounts added to the filler material and that which transfers to the smoke [1]. This is possibly due to reactions between ammonia compounds and other tobacco constituents. For example, diammonium phosphate reacts with sugars in the slurry process to form favorable flavor precursor compounds like deoxyfructazines [15–16], drastically reducing the amount of free ammonia available to transfer to the smoke. However, pyrolysis of nitrogenous compounds like amino acids and nicotine during smoking will produce detectable ammonia in the smoke [17].

The potential role of ammonia in both nicotine bioavailability and smoke toxicology make it an appropriate target for improved analytical measurement. The need to characterize the transfer of ammonia from filler to smoke and the opportunity to expand current methodologies to examine both the particulate and vapor phases of mainstream smoke led to the development of the analytical method described here. The improved analytical method to measure ammonia in tobacco filler was recently published [2]. The improved analytical method to measure ammonia in mainstream tobacco smoke with a Tedlar bag collection system in described herein.

## Experimental

### Sample Collection and Storage

Cigarette cartons were purchased locally and stored at room temperature prior to analysis. Before analysis, cigarettes packs were conditioned for at least 48 hours and no longer than 2 weeks at 22°C and 60% humidity [8]. Experiments measuring differences in ammonia content from packs opened vs. unopened during conditioning showed little difference in the ammonia in smoke measurement (data not shown), so samples designated for smoke analysis with conditioned with packs opened.

### Materials

An ISO Guide 34 endorsed 1000 mg/L ammonium standard was purchased from Sigma Aldrich (St. Louis, MO), Sulfuric acid (99%), falcon tube extraction vials; sample vials and volumetric flasks were purchased from Thermo Fisher (Waltham, MA). The ICS-3000 analytical system and columns were purchased from Thermo Scientific (Sunnyvale, CA). Tedlar bags were purchased from Supelco Inc. (Bellefonte, PA). 44mm Cambridge type fiberglass filter pads were purchased from Cerulean (Milton Keynes, England, UK).

### Sample Preparation

**Fig 1:**
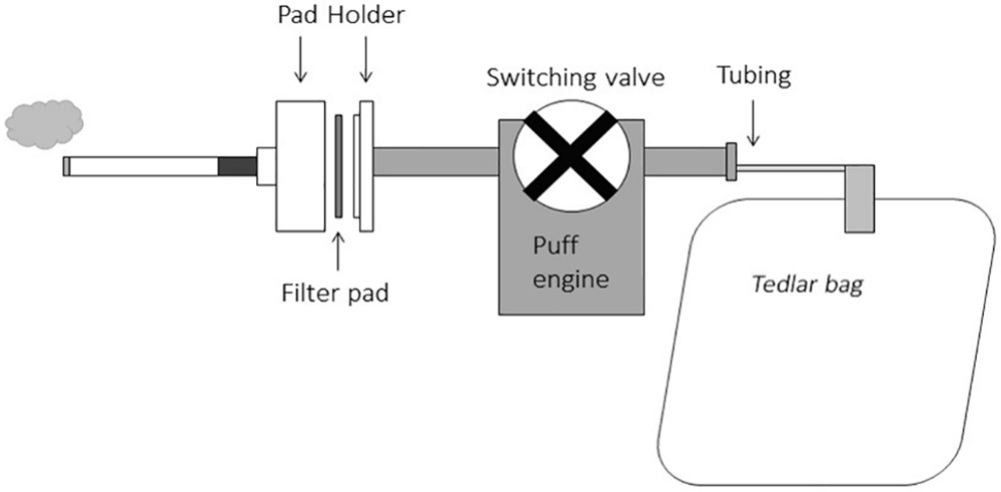
Pad + Bag mainstream smoke collection diagram.

Cigarettes were smoked on a Cerulean SM410 linear 10-port smoking machine (Cerulean, Milton Keyes, England, UK) according to ISO 3308 (35mL puff, 2 second duration, every 60 seconds) [18] or Canadian intense (CI) (55mL puff, every 2 seconds, every 30 seconds with ventilation holes blocked) conditions [19]. A 44mm Cambridge fiberglass filter pad (Whatman, Pittsburgh, PA) held in a pad holder was used to collect the particulate phase from the mouth end of the cigarette, while a 1L Tedlar gas sampling bag was used in place of the standard gas bag located downstream from the cigarette (Supelco, Bellefonte, PA) to collect the vapor phase (Fig 1) Pads were conditioned at 22°C and 60% relative humidity for at least 48 hours prior to smoking [20]. Tedlar bags are only used once and clean pad holders are used with each sample to minimize sample carryover and cross contamination. After smoking, the fiberglass pad was immediately placed in a 50mL falcon tube with 30mL 0.05M sulfuric acid extraction solution directly after removal from the smoking machine. The pad sample was shaken for 60 min at 160 rpm on a Barnstead/Labline Max Q 2000 shaker (Dubuque, IA). 10mL extraction solution was immediately added to the Tedlar bag through the valve opening directly after removal from the smoking machine and the bag samples were shaken on an Eberbach E6101 reciprocal shaker (Ann Arbor, MI) for 60 min at 180 osc/min. For both pad and bag samples, a 750 μL sample aliquot was centrifuged in a Sorval Pico Biofuge from Thermo Fisher (Waltham, MA) at 13,000 rpm for 2 min to remove debris. 700μL sample was added to a sample vial, vortexed and placed in a sample tray for analysis. Seven replicates were analyzed for each brand for both the ISO 3308 and Canadian intense smoking regimens. After smoking, 3 clearing puffs were used to ensure all smoke was in the Tedlar bag to minimize ammonia loss to tubing moisture.

### Instrumentation and Data Analysis

Samples were run on a Dionex ICS-3000 analytical system controlled by Chromeleon version 6.8 software (Thermo Scientific, Sunnyvale, CA). The autosampler injected 25μL of sample onto a 4 x 250 mm IonPac CS12A cation exchange column preceded by an IonPac CG12A guard column (4 x 50 mm) (Thermo Scientific, Sunnyvale, CA). Isocratic separation of the ammonium ion was achieved using a 20mM methanesulphonic acid (MSA) eluent prepared by the EG40 eluent generator (Thermo Scientific, Sunnyvale, CA) that required only a deionized water source. The column temperature was maintained at 25°C. The pumps and eluent generators were turned on at least 60 min before the first injection to allow baseline stabilization. Ammonium ions were detected using a conductivity detector. Prior to detection by the conductivity detector, the MSA eluent conductance was suppressed by the cation self-regenerating suppressor (CSRS). All ammonium values generated in Chromeleon were transferred to Microsoft Excel 2010 for further analysis. Statistical evaluations were done using JMP Software (SAS Institute Inc., Cary NC).

### Calibration and Quality Control

Calibration standards were made up in 25mL volumetric flasks with 0.05M sulfuric acid extraction solution and nine serial dilutions of the 1000 mg/L ammonium standard. For sample quantification, a 9-point standard curve was run daily with a calibration range of 0.05–10 mg/L. The calibration curves were very consistent, with the quadratic regression yielding an R2 value > 0.99. A typical batch includes a blank, 9 calibration standards, 1 quality control sample, and 9 unknown samples. The limit of detection, calculated using the Taylor method [21], was 15.6 μg/L, LOQ was 52 μg/L. Only data above LOQ were reported. The 3R4F research cigarette (University of Kentucky, Lexington KY) was selected as the QC material for the ammonia IC method and was included in each smoke and analytical run to ensure system integrity and reproducibility. Sample extracts were placed in -70°c freezers for long-term storage.

## Results and Discussion

### Development of Chromatographic Method

Isocratic separation for all abundant cation peaks (Li+, Na+, NH_4_+, K+, Mg+, and Ca+) was achieved with baseline resolution of the ammonium and other peaks at 20mM MSA concentration. Since ammonia and most simple ammonium salts are highly soluble in water, DI water should be more than sufficient to dissolve extractable ammonia from abundant sources. However, it was observed that using a 0.05M sulfuric acid extraction solution gave a better detector response with the lower ammonia concentrations measured in the Tedlar samples and was selected as the extraction solution for this method. In methods utilizing a mass spectrometer, an internal standard is typically used. Without such a detector, tracking instrument wellness can be challenging. For this method, we utilized morpholine, an analyte that elutes late on the CS12A column and does not interfere with any analytes of interest. While the morpholine response was not used to calculate ammonium amounts, it provided a way to track autosampler and detector performance. The peak height of morpholine was tracked over the course of the study, and stayed within 20% of the average throughout.

### Trapping Efficiency of the Cambridge Filter Pad

The Cambridge filter pad does not trap all ammonia in mainstream smoke, as ammonia is present in both the particulate and gas phase. Efforts to trap gas phase ammonia usually involve a series of impingers with an acidic trapping solution [22–24]. Impingers are costly, impede sample throughput and produce large amounts of chemical waste. In the development of this method, Cambridge filter pads were pre-treated with various acids and coupled with the use of a standard Tedlar bag gas phase collection system. To examine trapping efficiency, pads were pre-treated with ascorbic acid, citric acid, or glycolic acid and allowed to dry overnight. Samples were smoked using both the pre-treated pads and Tedlar bags to test ammonia breakthrough. Trapping efficiency by the pad was not substantially improved by acid pre-treatment as evidenced by all pre-treated pads still having significant ammonia breakthrough (>15%). This limitation in trapping efficiency was overcome by using a Tedlar bag to collect the gas phase ammonia. Combined with the Tedlar bags, treated or untreated pads provided excellent recovery, a faster sample throughput, and less chemical waste than previous impinger methods.

### Accuracy and Precision

Method accuracy was examined by spiking 4 μg/mL ammonium standard onto the Cambridge pad and into the Tedlar gas sampling bag (n = 7). A 3.79 μg/mL ammonia gas (Airgas, Atlanta, GA) was also spiked into separate Tedlar gas sampling bags (n = 7). Accuracy data are presented in Table 1. For this study, precision was defined as the relative standard deviation (RSD) of the QC material, a 3R4F research cigarette (S2 Table). Precision data are presented in Table 2. Our 3R4F values were consistent with values reported in the literature [23–26] (Table 3).

**Table 1:** Evaluation of Extraction Recovery using spiked standards for the ammonia in mainstream smoke method.

Spiked Standards	Spiked Into	Spike Amount (μg/mL)	Measured Amount	% Accuracy
**Ammonia Solution**	Pad extraction solution	4.00	3.47 ± 0.27	86.7 ± 6.7
**Ammonia Solution**	Tedlar bag extraction solution	4.00	4.2 ± 0.4	105 ± 11
**Ammonia Gas**	Tedlar bag	3.79	3.39 ± 0.20	89.5 ± 5.3

**Table 2:** Precision data for the ammonia in mainstream cigarette smoke method using the 3R4F reference cigarette.

Smoke Regime	ISO			CI		
Sample Component	Pad (μg/cig)	Bag (μg/cig)	Total Ammonia (μg/cig)	Pad (μg/cig)	Bag (μg/cig)	Total Ammonia (μg/cig)
**Average**	8.58	3.74	12.3	32.0	15.5	47.5
**SD**	1.14	1.03	1.9	4.1	2.3	5.4
**RSD**	13.3	27.5	15.4	12.8	14.7	11.4

**Table 3:** Comparison of 3R4F values to those reported in literature.

Authors	Collection Apparatus	Reported Values
**Baker et al. 2004 [23]**	Pad + 2 impingers with methanol and dilute nitric acid	16.0 μg/cig (1R4F)
**Mottier & Jeanneret 2011 [24]**	Pad + 1 impinger containing H2SO4	11.3 μg/cig (3R4F)
**Huang et al. 2003 [22]**	Pad + 1 impinger containing 0.005M HCL	16.2 μg/cig (1R4F)
**Callicutt et al [25]**	Pad + XAD-4 traps	7.1 μg/cig (1R4F)
**Counts et al. 2006 [26]**	Pad + impinger containing 10mM CH3SO3H	10.0 μg/cig (2R4F) 10.6 μg/cig (1R4F)
**Our Lab**	Pad + tedlar bag	13.0 μg/cig (3R4F)

### Ammonia Levels in Mainstream Tobacco Smoke

**Fig 2:**
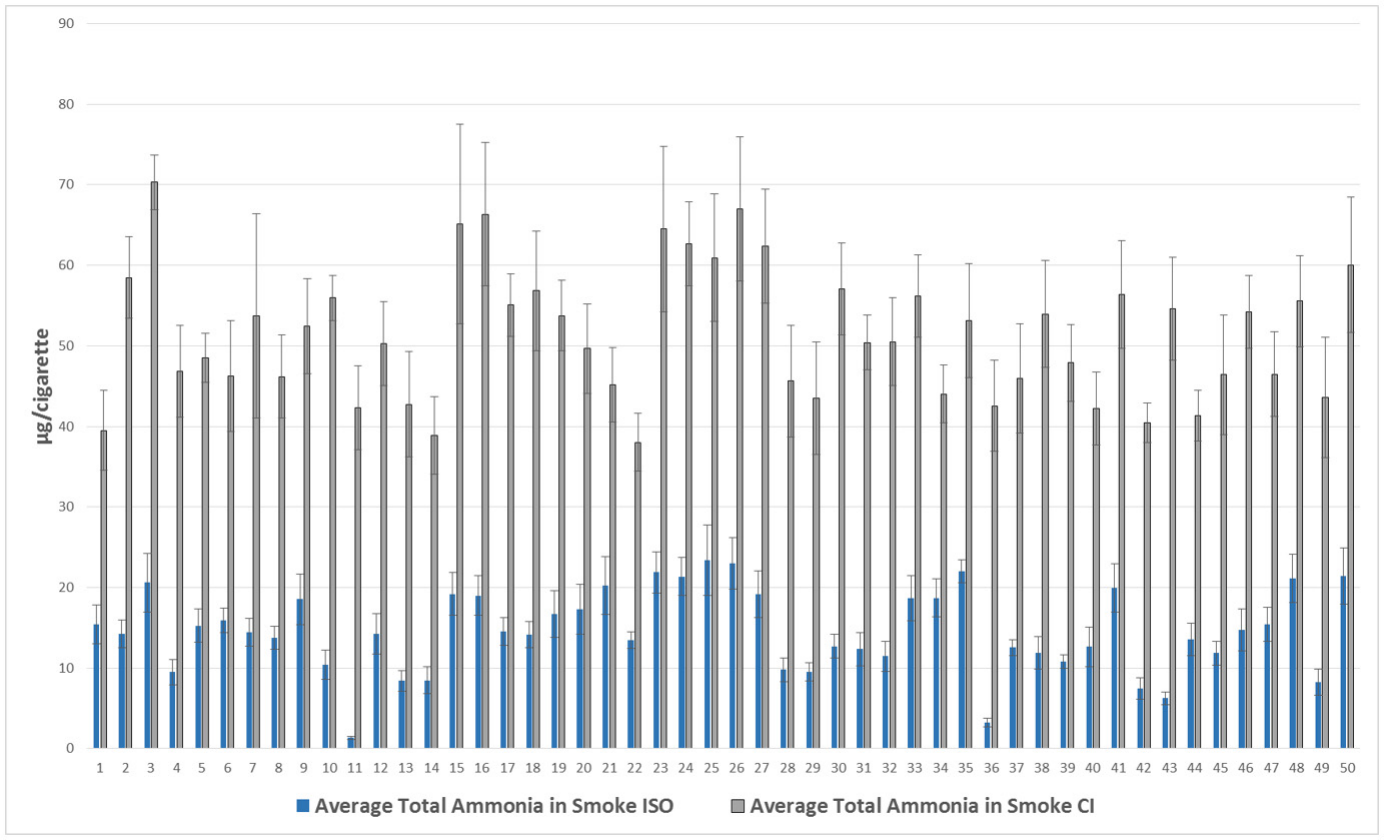
Average ammonia in mainstream cigarette smoke values (ISO and CI smoking conditions) for all commercial brands analyzed (n = 7).

The average total ammonia in mainstream smoke values from all commercial cigarettes brands ranged from approximately 1μg to 23μg per cigarette for ISO smoking conditions and 38μg to 70μg per cigarette for Canadian intense smoking conditions (Fig 2, S1 Table). Relative standard deviations (RSD) ranged from 6.2 to 21.6% for ISO smoking conditions and 5.1 to 19% for CI smoking conditions, with highly ventilated cigarettes having higher RSD’s for within brand replicate measurements (n = 7). Philip Morris brands had the highest average ammonia delivery for both smoking regimens, but there was little variation in all other manufacturers. As expected, under ISO conditions, with increasing tip ventilation, there is a decrease in ammonia smoke values. When smoked under CI conditions, no trend was observed, as ventilation holes are blocked under this regime. When cigarettes are grouped by FTC tar delivery, there is a statistically significant difference in ammonia smoke deliveries between cigarettes formerly marketed as full flavor, light and ultra-light, with full flavored cigarettes delivering the highest amount of ammonia.

When examining percent breakthrough, defined as the percent of total ammonia found in the Tedlar bag, statistically significant differences were also observed. Under ISO conditions, percent breakthrough was the highest for cigarettes formerly marketed as “ultra-light,” followed by “light” and full flavored cigarettes. Under CI conditions, there are no statistically significant differences in breakthrough by these categories. These findings could indicate that the increased tar and moisture present in the Cambridge filter pad under CI smoking conditions traps some ammonia during smoking and has implications for smoke methods measuring other volatile components. If a Cambridge filter pad acts as a stationary phase, methods employing a headspace extraction directly above the pad may result in values (e.g., free nicotine analyses) lower than the amount actually present. Increased moisture in the cigarette filter could also trap ammonia before it reaches the pad. Data collected during method development indicates that a significant amount of ammonia is effectively trapped in the filter, which in part explains the apparent lower rate of transfer of ammonia from tobacco filler to smoke. Ammonia trapped in the tubing connecting the smoking port to the Tedlar bag, was not measured and is a limitation of this study.

Our values show there is a vast difference between the amount of ammonia added to filler and the amount making its way to mainstream smoke. In fact, using previously published filler data [2], the percent transfer from filler to smoke for all 50 brands analyzed ranged from 0.1% to 2.1% for ISO conditions and 1.8% to 5.3% for CI. These results are not surprising. As stated previously, ammonia is expected or intended to react with other chemicals in the tobacco filler before transferring to the smoke [7,12,27]. Reacting sugars with DAP creates deoxyfructazines which then pyrolize to produce weak bases such as pyrazines and pyridines present in the smoke [15–16]. These compounds, along with ammonia and alkaloids, were included in a total volatile bases (TVB) measurement found in older industry documents [28–29]. An additional percentage is trapped by the filter and lost to side stream smoke.

## Conclusion

We developed a faster, less chemically cumbersome method for determining ammonia in mainstream smoke. This method provides excellent sensitivity and selectivity for ammonia in the particulate and vapor phases. Data previously reported [2] and that presented here further shows there is a large difference between ammonia measured in the filler and that found in smoke. It is beyond the scope of this analytical method to draw correlations between ammonia concentrations in mainstream smoke and free nicotine or to determine if ammonia is the primary driver of smoke alkalinity. The goal of this work was to develop a rugged and high throughput method for making reliable measurements of mainstream smoke ammonia deliveries. This was accomplished using standard smoking protocols, with only a slight modification of replacing the standard gas sampling bag with a clean Tedlar bag, and ammonia in both the vapor and particulate phase were measured to determine the total ammonia delivery. The advantages of this new approach include the ability to accurately measure products with a wider range of ammonia deliveries with high throughput while minimizing solvent waste.

## Supporting Information

S1 Table Average ammonia in mainstream cigarette smoke data (ISO and CI smoking conditions) for 50 commercial brands analyzed (n = 7). (XLSX)

S2 Table QC characterization data for the 3R4F research cigarette. (XLSX)
